# Comparison of Outcomes Between a Fixed-Loop Cortical Suspensory Device and Metal Interference Screw for Femoral Fixation of Bone–Patellar Tendon–Bone Grafts in Anterior Cruciate Ligament Reconstruction: A Scandinavian Registry Study

**DOI:** 10.1177/23259671251335978

**Published:** 2025-05-22

**Authors:** Alexander Nagel Tandberg, Marek Zegzdryn, Cathrine Aga, Stein Hakon Lastad Lygre, Tone Gifstad, Jon Olav Drogset, Lars Engebretsen, Martin Lind, Magnus Forssblad, Stig Heir

**Affiliations:** † Department of Orthopedic Surgery, Akershus University Hospital, Akershus, Norway; ‡ Department of Orthopedic Surgery, Martina Hansens Hospital, Bærum, Norway; § Department of Orthopedic Surgery, Sørlandet Hospital, Arendal, Aust-Agder, Norway; ‖ Department of Occupational Medicine, Haukeland University Hospital, Bergen, Hordaland, Norway; ¶ Norwegian Knee Ligament Register, Orthopaedic Surgery, Haukeland University Hospital, Bergen, Hordaland, Norway; # Orthopaedic Surgery, Trondheim University Hospital, Trondheim, Trøndelag, Norway; ** Oslo Sport Trauma Research Center, Norwegian School of Sport Sciences, Oslo, Norway; †† Orthopeadic Division, University of Oslo, Oslo, Norway; ‡‡ Department of Orthopaedics, Aarhus University Hospital, Aarhus, Denmark; §§ Department of Molecular Medicine and Surgery, Stockholm Sports Trauma Research Center, Stockholm, Sweden; Investigation performed at Department of Orthopedic Surgery, Martina Hansens Hospital, Bærum, Norway

**Keywords:** ACL, BPTB, cortical fixation, interference screw fixation

## Abstract

**Background:**

Use of a cortical suspensory device (CSD) is an alternative fixation method for bone–patellar tendon–bone (BPTB) in the femur. To the authors’ knowledge, no study has compared revision outcomes and patient-reported outcomes (PROs) with the traditional interference screw (IS) fixation.

**Purpose:**

To compare the survival rate of grafts fixated on the femoral side with either a fixed-loop CSD or IS for primary anterior cruciate ligament reconstructions (ACLRs) with BPTB autograft 2 and 5 years after index surgery, as well as to compare PROs between the same 2 treatment groups based on data from 3 National Knee Ligament Registers (NKLRs) in Scandinavia.

**Study Design:**

Cohort study; Level of evidence, 2.

**Methods:**

The study included patients registered in the NKLRs of Norway, Denmark, and Sweden who underwent primary ACLR from 2005 to 2021. Patients undergoing additional surgery for concomitant injuries to the posterior cruciate ligament, lateral collateral ligament, or medial collateral ligament and patients with major cartilage lesions were excluded. Cox regression models with and without adjustment for possible confounding from age, sex, meniscal and chondral injury, tibial fixation, surgical time, time from injury to surgery, injury mechanism, and country were used to calculate hazard ratios (HRs) and 95% confidence intervals. The possible effects of femoral fixation on 1-year postoperative KOOS QoL and on change from preoperative to 1-year postoperative KOOS QoL were analyzed using multiple linear regression with adjustment from possible confounding from the same variables. *P* values <.05 were considered significant.

**Results:**

A total of 13,955 primary ACLRs met the inclusion criteria during the study period; 1852 patients (13%) had a femoral CSD fixation and 12,103 patients (87%) a femoral IS fixation. No significant difference was observed in overall risk of revision between CSD and IS (HR, 1.10; 95% CI, 0.83 to 1.47; *P* = .5), and differences in cumulative survival at 2 years (CSD: 98.3% vs IS: 98.6%) and 5 years (CSD: 96.3% vs IS: 96.4%) were not statistically significant. No difference was found for mean KOOS QoL delta (1 year minus baseline) when adjusting for possible confounders (mean difference, –1.60; 95% CI, –3.83 to 0.63; *P* = .16).

**Conclusion:**

Data from the Scandinavian NKLRs showed no difference in the risk of revision and no difference in the KOOS QoL for fixed-loop CSD fixation compared with IS fixation on the femoral side with BPTB grafts in primary ACL reconstruction.

Primary anterior cruciate ligament reconstruction (ACLR) is a common orthopaedic operation among young and active individuals.^13^ Bone–patellar tendon–bone (BPTB) autografts are now the most frequent graft in primary ACLR in Norway.^39^ However, the risk of revision is still high in the younger population,^21,28^ and the functional results are variable, with only half of the patients returning to preinjury levels of sport after surgery.^8^ In addition, surgically treated patients have a risk of developing long-term osteoarthritis in the knee.^18^

Correct graft positioning and fixation are crucial factors for receiving a successful reconstruction and proper knee kinematics and function.^17^ Interference screw (IS) has been considered the gold standard when fixating the femoral side of BPTB autografts in primary ACLR.^10,37^ However, the threads of the IS may cause graft damage.^24^ Bone block breakage due to the screw and tendon avulsion from the bone block are other potential risk factors when using an IS.^26^

An alternative femoral fixation method for BPTB graft fixation is using a cortical suspensory device (CSD).^26^ While femoral CSD is a widely used fixation method for soft tissue grafts and has been used for many years, the corresponding device for BPTB grafts has only been used in 10% to 12% of primary ACLRs.^34,37^ Both fixed- and adjustable-loop suspensory systems are commercially available.^14,26,32^ While both systems show desirable biomechanical properties, fixed suspensory systems have been shown to be biomechanically superior, with a more consistent and reproducible result compared with adjustable suspensory systems.^3^ The clinical results after femoral CSD with BPTB are promising with regard to patient-reported outcomes (PROs),^11,32,36,40^ return to play,^11,32,40^ and healing of the bone block^11,23,36,38,40^ within the femoral tunnel. However, tunnel widening^5^ and graft migration,^26,36^ and eventually the risk of graft failure, might be of concern when using a CSD.

To our knowledge, no study has so far evaluated revision outcome and PROs in primary ACLR with BPTB autograft comparing femoral fixed-loop CSD with femoral metal IS fixation. Moreover, because of the relatively recent development and infrequent use of femoral CSD with BPTB autografts in primary ACLR, no cohort study has reported on the survival rate of this fixation method.

The purpose of this study was to compare the risk of revision and survival rate of grafts fixated with the Endobutton CL BTB Fixation System (Smith & Nephew; hereinafter referred to as CSD) to the traditional metal IS for primary ACLR with BPTB autograft based on data from the National Knee Ligament Registers (NKLRs) in Scandinavia. The secondary aim was to investigate the PROs between the same 2 treatment groups.

## Methods

The study included prospectively collected data on patients from the NKLRs of Norway, Denmark, and Sweden who underwent primary ACLR. Prospectively collected data from the 3 Scandinavian registries were pooled for analyses comparing the CSD with the IS. Approval for utilization of the register data was applied for through the Regional Committees for Medical Research Ethics–South East Norway (REK reference No. 366407). No written consent is necessary in Denmark for studies based on data from national board of health–approved national health care registries. For participants in Norway and Sweden, registration is voluntary. The respective boards of the NKLRs were informed and approved the study.

### Scandinavian NKLRs

The 3 Scandinavian NKLRs cover >20 million people. Data on patient characteristics, injury pathology, surgical technique, additional injury of the knee, and the fixation devices are recorded.^2,13,19^ Any revision is reported to the registers by the surgeon. In addition, the registers collect PROs from the patients in the form of the Knee injury and Osteoarthritis Outcome Score (KOOS). Close to 100% completeness has been found when comparing primary ACLRs in the Norwegian NKLR with hospital protocols and the Norwegian Patient Registry.^13^ In Denmark, 88% of all ACLRs are reported in the registry,^10^ while the Swedish registry has >90% completeness.^2^ Electronic registration forms are now available in all 3 countries.^39^

### Patients and Patient Characteristics

Inclusion criteria were primary ACLR with BPTB autograft with femoral fixation using a fixed CSD or metal IS, performed during the inclusion period. The inclusion period was from the introduction of the CSD to the registries (2012 in Norway, 2005 in Sweden, and 2006 in Denmark) until end of 2021. Patients aged 15 to 59 years at time of surgery were included. Exclusion criteria were ACLRs with other graft types than BPTB autograft, fixation with nonmetal IS, major cartilage lesions requiring surgery, or reconstruction of the posterior cruciate ligament, lateral collateral ligament, or medial collateral ligament performed on the same knee. No censoring was done because of emigration or death.

### Outcomes

The risk of revision and survival rate of primary ACLR at 2 years after surgery, compared between the IS and CSD groups, were considered as the primary outcomes. Revision was defined as the insertion of a new ACL graft in a primary ACL-reconstructed knee. Secondary outcomes were survival rate at 5 years and the KOOS QoL^31^ at 1 (Denmark and Sweden) and/or 2 (Norway and Sweden) years, comparing the 2 groups. KOOS QoL and Sport and Recreation are the most responsive KOOS subscales at follow-up after ACLR.^2^ KOOS QoL was chosen in this study because it had the greatest response rate in all 3 registries.

### Statistical Analysis

A combined file with aggregated data from all 3 countries was obtained and processed in the SPSS Statistics software program (IBM Corp) for analysis. All reconstructions that met the inclusion criteria were included. The survival estimates at 2 and 5 years and the cumulative survival were detected in each group using a Kaplan-Meier survival table. Cox regression analysis was performed, and the possible effect on risk of revision from femoral fixation was analyzed using Cox regression analysis with adjustment for possible confounding from sex, age in years (<20, 20-24, 25-29, 30-45, or >45), meniscal or chondral injury (yes/no), fixation device utilized on the tibial side, surgical time, time from injury to surgery, pivoting activity, and registry by nation. The outcome was expressed with hazard ratios (HRs) and their 95% confidence intervals and the respective *P* value. The possible effects of femoral fixation on 1-year postoperative KOOS QoL and on change from preoperative to 1-year postoperative KOOS QoL were analyzed using multiple linear regression with adjustment from possible confounding from the same variables. *P* values <.05 were considered significant.

## Results

**Figure 1.**
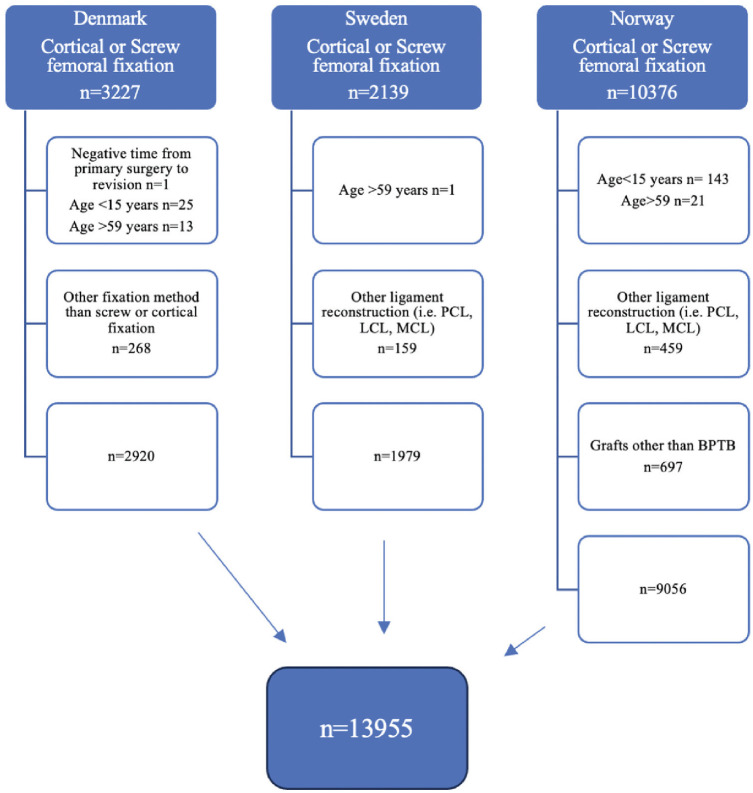
Flowchart of primary anterior cruciate ligament reconstructions with bone–patellar tendon–bone grafts and femoral fixation with the cortical suspensory device or interference screw from the National Knee Ligament Registers of Norway, Denmark, and Sweden. LCL, lateral collateral ligament; MCL, medial collateral ligament; PCL, posterior cruciate ligament.

A total of 13,955 primary ACLRs met the inclusion criteria during the study period (Figure 1); 1852 patients (13%) had a femoral CSD fixation and 12,103 patients (87%) a femoral IS fixation. Norway had the largest number of primary ACLRs with BPTB grafts (9056 patients), followed by Denmark (2920 patients) and Sweden (1979 patients). Baseline patient and surgical characteristics for the 2 fixation groups are presented in Table 1.

**Table 1:** Patient Characteristics of the 2 Fixation Groups^*a*^

	Screw Fixation	Cortical Fixation	*P* ^*b*^
No. of knees	12,103	1852	
Mean age, y (range)	28 (15-59)	25 (15-58)	<.001
Female sex	39	47	<.001
Meniscal injury	51	60	<.001
Chondral injury	21	16	<.001
Mean surgical time, min	78	65	<.001
Mean time from injury to surgery, days	604	375	<.001
Pivoting activity	60	69	<.001
Metal screw fixation in tibia	81	79	<.001

a Data are presented as percentage unless otherwise indicated.

b The *t* test was used for continuous variables and the chi-square test for categorical variables.

**Figure 2.**
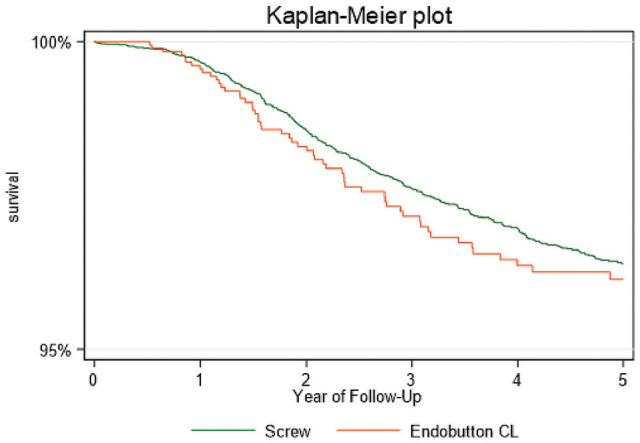
Kaplan-Meier survival plot showing the cumulative graft survival for interference screw and cortical suspensory device (Endobutton CL BTB fixation system) femoral fixation of bone–patellar tendon–bone grafts in primary anterior cruciate ligament reconstructions.

We did not observe any significant difference in overall risk of revision between CSD and IS (HR, 1.10; 95% CI, 0.83-1.47; *P* = .5), and differences in cumulative graft survival at 2 years (CSD: 98.3% vs IS: 98.6%) and 5 years (CSD: 96.3% vs IS: 96.4%) were not statistically significant. Survival rates at 2 and 5 years after the index surgery are presented in Table 2 and as Kaplan-Meier survival estimates in Figure 2.

**Table 2:** Cumulative Survival Estimates at 2 and 5 Years and Crude and Adjusted HR^*a*^

	n (%)	2-y Survival, %	5-y Survival, %	Median Follow-up, y	Crude HR	95% CI	*P*	Adjusted HR^*b*^	95% CI	*P*
Fixation femur										
Screw fixation	12,103 (86.7)	98.6	96.4	6.60	1			1		
Cortical fixation	1852 (13.3)	98.3	96.3	4.34	1.19	0.91-1.55	.2	1.10	0.83-1.47	.5
Fixation femur (0-5 y)^*c*^										
Screw fixation	12,103 (86.7)			5.00	1			1		
Cortical fixation	1852 (13.3)			4.34	1.08	0.81-1.45	.6	1.02	0.74-1.39	.9
Fixation femur (>5 y)^*c*^										
Screw fixation	7035 (90.5)			7.13	1					
Cortical fixation	735 (9.5)			1.38	2.22	1.09-3.83	.03	1.69	0.86-3.31	.1

a HR, hazard ratio.

b Adjusted for sex, age, meniscal injury, cartilage injury, tibia fixation material, country, time since injury, surgical time, and activity.

c Analysis done at separate time periods to fulfill assumption of proportional hazard.

The KOOS QoL values from the 2 fixation groups are presented in Table 3. No difference was found for mean KOOS QoL delta (1 year minus baseline) when adjusting for possible confounders (mean difference, –1.60; 95% CI, –3.83 to 0.63; *P* = .16) (Table 4). The completeness of KOOS QoL for all 3 registries was 66% preoperatively and 46% at the 1-year follow-up.

**Table 3:** KOOS QoL at Baseline, at 1 After Surgery, and Delta^*a*^

	Screw Fixation	Cortical Fixation	*P* ^*b*^
Mean KOOS QoL at baseline	35.7 (35% missing)	38.4 (27% missing)	<.001
Mean KOOS QoL at 1 y	63.3 (54% missing)	66.3 (46% missing)	<.001
Mean KOOS QoL delta^*c*^	28.2	28.2	.9

a KOOS QoL, Knee injury and Osteoarthritis Outcome Score Quality of Life

b The *t* test was used for continuous variables and the chi-square test for categorical variables.

c Delta is change from baseline to 1 year.

**Table 4:** KOOS QoL for Screw Fixation Compared With Cortical Fixation at 1 Year and Delta^*a*^

	Crude			Adjusted^*b*^		
	Mean Difference	95% CI	*P*	Mean Difference	95% CI	*P*
KOOS QoL at 1 y						
Screw fixation	—			—		
Cortical fixation	2.96	1.30 to 4.61	<.001	0.09	−1.64 to 1.83	.9
KOOS QoL Delta^*c*^						
Screw fixation	—			—		
Cortical fixation	0.04	−2.08 to 2.16	.97	−1.60	− 3.83 to 0.63	.16

a KOOS QoL, Knee injury and Osteoarthritis Outcome Score Quality of Life

b Adjusted for sex, age, country, meniscal injury, cartilage injury, tibia fixation, pivoting activity, time from injury to surgery, and surgical time.

c Delta is change from baseline to 1 year (Denmark and Sweden) or 2 years (Norway).

## Discussion

The primary finding of the present study was that there was no significant difference in the risk of ACL revision between the fixed-loop CSD and IS for femoral fixation of autologous BPTB graft in primary ACLR at 2 years. Secondarily, there was no significant difference in the risk of revision at 5 years. This is the first registry-based study comparing the 2 fixation methods for BPTB grafts.

One retrospective study has previously evaluated revision rates of BPTB using CSD for femoral fixation. Barret et al^4^ reported a clinical failure in 3 of 37 patients with a median follow-up of 52 months. One of the patients who underwent revision, while the other 2 were treated with a brace. Feller and Webster^11^ reported 1 rerupture of 31 patients who underwent ACLR using BPTB and femoral fixation with CSD during the follow-up time. The rerupture occurred during a high-risk activity 6 months postoperatively. A the 15-year follow-up, a study reporting on the same patient cohort showed no additional reruptures.^40^ Taketomi et al^36^ reported zero reruptures 1 year postoperatively in 34 patients with BPTB and femoral fixation with CSD. Sanada et al^32^ evaluated 27 young female athletes who underwent ACLR with BPTB autograft with femoral fixation using CSD. No reruptures in the BPTB group were observed with a mean follow-up of 34 months.

The use of CSD fixation for BPTB grafts in ACLR in the femur may offer advantages over traditional IS fixation. The bone block is fully emersed into the femoral socket, which may give a theoretical biological advantage with respect to healing.^33^ CSD may be helpful in cases of graft-tunnel mismatch and in cases of posterior wall blowout.^6^ Graft displacement and screw damage of passing sutures or tendon are also not of concern.^24^ Lastly, in revision cases, CSD fixation is associated with less risk of one needing bone grafting, compared with IS fixation.^22^ The drawbacks of CSD fixation are the potential lack of integration to the adjacent tunnel wall,^23^ tunnel widening,^5^ button rotation or soft tissue interposition,^36^ graft migration^26,36^ or subsidence, and eventually the risk of graft failure.

The KOOS QoL was chosen as a main secondary outcome because it had the greatest response rate in all 3 registries. The KOOS is a validated PRO for patients undergoing ACLR. The KOOS Sports and Recreation and KOOS QoL subscales are considered to be the most responsive during postoperative follow-up after ACLR.^31^

In the present study, there was no difference in postoperative adjusted KOOS QoL at follow-up or change in KOOS QoL from baseline (Tables 3 and 4). Only 1 previous study has compared the clinical outcome of the 2 fixation methods and BPTB graft using PROs. Jeon et al^15^ found a 10-point difference in Lysholm score in favor of IS fixation postoperatively. This was nonsignificant, however, and the study was limited by its small sample size.

One important strength of this study is the large sample size of patients. With novel devices, it is of particular importance to follow them in large prospective registry-based studies to be able to detect devices with inferior outcomes early.^9^

### Limitations

Registry data do have limitations. The 2 fixation groups presented some differences in patient characteristics at baseline (Table 1). Sex,^27^ age,^7,35,41^ the presence of a meniscal or chondral injury at the index operation,^12^ the fixation device utilized on the tibial side,^1,25^ and the time from injury to surgery^7^ may all affect the risk of revision in patients with ACLR. Thus, the results are number adjusted through regression analysis.

In Denmark and Sweden, hamstring tendon autografts are the graft of choice in most primary ACLRs.^16,30^ Hence, in these countries surgeons may use BPTB autografts for a selected group of patients. Moreover, using revision surgery as an endpoint has its limitations as well. First, not all patients experiencing rerupture will undergo revision surgery. Second, revision is largely performed due to increased laxity and giving away. Thus, revision rates do not necessarily reflect the number of patients with a poor clinical outcome. Besides, not all variables known to affect the outcome of ACLR are registered in the NKLRs. Body mass index, for instance, which is considered a confounder,^7,35^ was not included in the final analysis because weight and height are not registered in the Danish registry. Other limitations to note are lack of data on surgeon volume, which has not been shown to affect revision rate in other registry studies.^20^

Finally, the response rate for KOOS was low, and the results must be interpreted with care. Less than half of the patients in this study responded to the KOOS at 1 or 2 years postoperatively. However, a validation study from the Danish Knee Ligament Reconstruction Registry showed no difference in any of the 5 KOOS subscales between responders and nonresponders.^29^

## Conclusion

Data from the Scandinavian NKLRs showed no difference in the risk of revision and no difference in the KOOS QoL for a fixed-loop CSD fixation compared with IS fixation on the femoral side with BPTB grafts in primary ACLR.
